# Development and Validation of a Rapid LC-MS/MS Method for Quantifying Eravacycline in Epithelial Lining Fluid: Application to a Prospective Pulmonary Distribution Study in HAP/VAP Patients

**DOI:** 10.3390/antibiotics14090957

**Published:** 2025-09-22

**Authors:** Jingjing He, Jingjing Lin, Xin Li, Nanyang Li, Jianguang Su, Jufang Wu, Jin Hu, Jing Zhang, Xiaofen Liu

**Affiliations:** 1 Key Laboratory of Clinical Pharmacology of Antibiotics, Institute of Antibiotics, National Health Commission, National Clinical Research Center for Aging and Medicine, Huashan Hospital, Fudan University, Shanghai 200040, China; 2 Clinical Pharmacology Center, Huashan Hospital, Fudan University, Shanghai 200040, China; 3 Department of Clinical Nutrition, Huashan Hospital, Fudan University, Shanghai 200040, China; 4 Department of Neurosurgery, Huashan Hospital, Fudan University, Shanghai 200040, China; hujin@fudan.edu.cn

**Keywords:** LC-MS/MS, eravacycline, epithelial lining fluid, pulmonary penetration, HAP/VAP

## Abstract

**Background**: Eravacycline exhibits potent activity against multidrug-resistant pathogens and holds promise for the management of hospital-acquired and ventilator-associated pneumonia (HAP/VAP). However, sensitive and robust bioanalytical methods to quantify eravacycline in human pulmonary epithelial lining fluid (ELF) for pharmacokinetic (PK) and pulmonary penetration studies in these infections remain limited. **Methodology**: A simple, rapid, and sensitive LC-MS/MS method was developed for the quantification of eravacycline in bronchoalveolar lavage fluid (BALF). Using urea as a volume normalizer, ELF concentrations were calculated from the eravacycline concentrations in BALF. This method was applied in a clinical study evaluating the pulmonary penetration after intravenous infusion in patients with HAP and VAP. **Results**: The developed LC-MS/MS method exhibited good linearity in the range of 1–200 ng/mL for quantifying eravacycline in BALF. In BALF, intra-day precision ranged from 1.4% to 6.0%, and inter-day precision from 1.6% to 9.9%, with accuracy between 98.0% and 102.4%. Matrix effects were within 97.4% to 107.6% for BALF samples from six different individuals, with extraction recoveries ranging from 103.5% to 107.2%. Stability studies demonstrated that eravacycline remained stable under various conditions, including storage at room temperature, freeze–thaw cycles, long-term (–70 °C) storage, and post-treatment handling. The method was successfully applied to clinical samples from four HAP or VAP patients, with measured eravacycline pulmonary penetration ratios of 4.29, 17.40, 5.22 and 4.70, indicating efficient pulmonary distribution. The measured eravacycline concentrations ranged from 0.0243 to 0.0436 μg/mL in BALF. The corresponding urea-corrected ELF concentrations ranged from 0.570 to 1.617 μg/mL. **Conclusions**: This study described a detailed and validated method for quantifying eravacycline concentrations in ELF from patients, providing a reliable analytical approach for investigating the pulmonary distribution of eravacycline.

## 1. Introduction

Hospital-acquired pneumonia (HAP) and ventilator-associated pneumonia (VAP) are among the most common causes of hospital-acquired infections, particularly in intensive care units (ICU), and represent major causes of morbidity and mortality in critically ill patients [1,2,3,4]. HAP/VAP can be caused by multidrug-resistant (MDR) strains of Gram-negative bacteria, with MDR pathogens accounting for approximately 64.9% of cases, including MDR *Pseudomonas aeruginosa* and *Enterobacterales* species (such as *Klebsiella pneumoniae* and *Escherichia coli*) *and* carbapenem-resistant *Acinetobacter baumannii* (CRAB) [5,6,7]. Moreover, MDR infections are increasing in prevalence with high mortality and morbidity rates due to limited treatment options and delays in commencing suitable treatment [8,9].

In response to this growing challenge, eravacycline has emerged as a promising therapeutic option. It is a novel, fully synthetic fluorocycline antibiotic with potent in vitro activity against a broad spectrum of pathogens, including MDR and extensively drug-resistant (XDR) Gram-negative bacteria such as carbapenem-resistant *Enterobacteriaceae* (CRE) and CRAB [10,11,12,13]. It has been approved for the treatment of complicated intra-abdominal infections (cIAI) in adult patients [14,15]. Early pharmacokinetic (PK) studies of eravacycline in healthy volunteers have demonstrated favorable pulmonary penetration, including into epithelial lining fluid (ELF), suggesting its potential clinical utility in treating respiratory infections such as HAP and VAP [16]. However, the PK characteristics in critically ill HAP/VAP patients may differ due to altered physiology, ongoing inflammation, concomitant medication and organ dysfunction [17]. To date, no data on the pulmonary penetration of eravacycline in patients with HAP/VAP have been reported.

To better understand these potential differences, we are conducting a prospective clinical study (registered in chictr.org.cn as ChiCTR2400087137) to evaluate the PK characteristics and pulmonary penetration of eravacycline in patients with HAP/VAP. As a critical component of this investigation, accurate quantification of eravacycline concentration in biological matrices, such as human plasma and ELF, is essential for generating reliable pharmacokinetic/pharmacodynamic (PK/PD) data and supporting optimized dosing strategies prior to larger-scale trials [18,19]. ELF represents a key compartment for assessing drug exposure at the site of pulmonary infection, directly bathing the airway and alveolar surfaces where causative pathogens colonize [20]. Antibiotic concentrations in ELF are critical for efficacy against extracellular organisms, and the ELF-to-plasma concentration ratio is commonly used as an indicator of pulmonary drug penetration and serves as an important parameter for optimizing dosing strategies in pneumonia treatment [21]. Currently, LC-MS/MS methods for quantifying eravacycline in ELF are limited. To date, only one published PK study in healthy participants has referred to a bioanalytical method for measuring eravacycline concentrations in ELF using BALF as the sampling matrix [16]. In that study, plasma underwent solid-phase extraction, whereas BALF samples were acidified with 0.1% trifluoroacetic acid (TFA) prior to analysis without further extraction. Critically, this bioanalytical method was developed and validated using samples predominantly derived from healthy or non-infected participants. However, BALF from patients with HAP/VAP constitutes a significantly more complex biological matrix. This complexity arises from elevated levels of inflammatory cells, cytokines, proteins, cellular debris, bacteria and other pathological constituents, which may introduce substantial matrix effects [22]. Consequently, analytical methods optimized solely for healthy volunteer BALF are likely to yield suboptimal performance when applied to patient-derived samples. It is thus critical to develop and validate bioanalytical methods specifically tailored to the complex matrix of patient-derived ELF, with improved sensitivity and robustness to reliably support PK evaluations in clinical settings.

In this study, we developed a simple, specific and reproducible LC-MS/MS method with high sensitivity for the quantification of eravacycline in ELF. The method was validated in accordance with the M10 bioanalytical guidelines and was successfully applied in our clinical study to quantify eravacycline concentrations in ELF of four patients with HAP or VAP. To the best of our knowledge, this is the first report detailing a validated method for determining eravacycline concentrations in ELF in critically ill HAP/VAP patients, providing a practical analytical tool for future pulmonary PK/PD studies.

## 2. Materials and Methods

### 2.1. Chemicals and Reagents

The reference standard of eravacycline (purity: 79.5%) was produced by Changzhou SynTheAll Pharmaceutical Co., Ltd. (Changzhou, China). The internal standard [^2^H_8_]-eravacycline (purity: 97.14%) was supplied by Wuhan WuXi AppTec (Wuhan, China). [^15^N_2_]-urea, [^13^C,^15^N_2_]-urea, acetonitrile (HPLC grade), methanol (analytical grade), TFA (HPLC grade), and formic acid (FA, analytical grade) and urea were all purchased from Sigma Aldrich (St. Louis, MO, USA). Ultrapure water was obtained using the Millipore ultrapure water system (Millipore, Billerica, MA, USA).

### 2.2. LC-MS/MS Method

The quantitative analysis was performed on a Sciex API 5500 triple quadrupole mass spectrometer (AB Sciex, Framingham, MA, USA) coupled with a Shimadzu LC-30A UPLC system (Shimadzu Corporation, Kyoto, Japan) platform. To perform the separation of eravacycline, an ACQUITY UPLC BEH C18 column (1.7 μm, 2.1 × 50 mm) was employed, and column temperature was set at 40 °C. The mobile phase system consisted of 0.3% FA in acetonitrile as mobile phase A and 0.3% FA in water as mobile phase B. The gradient elution was carried out at a constant flow rate of 0.4 mL/min, with the following gradient of mobile phase A and B: 0% A held from 0.0 to 0.1 min, linearly increased to 25% A at 1.6 min, then rapidly increased to 90% A at 1.9 min, maintained at 90% A until 2.5 min, and finally returned to the initial condition of 10% A at 2.6 min, with a total run time of 3.5 min. The autosampler was set at 4 °C with an injection volume of 5 μL.

Mass spectrometry was performed using electrospray ionization in positive mode with the following optimized parameters: an ion spray voltage of 4000 V, a source temperature of 550 °C, curtain gas (nitrogen) at 35 psi, nebulizing gas (GS1, nitrogen) at 60 psi, auxiliary gas (GS2, nitrogen) at 60 psi, and collision gas (nitrogen) set to 8 psi. The analytes were monitored in multiple reaction monitoring (MRM) mode using their mass-to-charge ratios (*m*/*z*) with the transition’s m/z 559.2 → 542.2 for eravacycline and *m*/*z* 567.4 → 550.2 for the internal standard ([^2^H_8_]-eravacycline). Data acquisition and processing were performed using Analyst software (version 1.6.2). Quantification based on peak area ratios using linear regression with 1/*x*^2^ weighting, and regression analysis was performed by Watson LIMS (version 7.5).

### 2.3. The Preparation of Calibration Curve, Quality Control and Internal Standard

Eravacycline reference standard was accurately weighed and dissolved in methanol containing 0.1% TFA to prepare standard stock solution and quality control (QC) stock solutions at a concentration of 1000 μg/mL. These stock solutions were subsequently diluted with 50% methanol solution containing 0.1% TFA to generate calibration curves and QC working solutions at appropriate concentrations. For BALF calibrators, working stock solutions were prepared at concentrations of 0.02, 0.04, 0.1, 0.2, 0.5, 1, 2, and 4 μg/mL, corresponding to final calibration concentrations of 1, 2, 5, 10, 25, 50, 100, and 200 ng/mL. For BALF QC samples, working stock solutions were prepared at concentrations of 0.06, 1.2, and 3 μg/mL, corresponding to QCL, QCM, and QCH concentrations of 3, 60, and 150 ng/mL, respectively.

Additionally, [^2^H_8_]-eravacycline internal standard was accurately weighed and dissolved in methanol containing 0.1% TFA to prepare a stock solution at 1000 μg/mL. This stock was then diluted with pure methanol to obtain an IS working solution at a final concentration of 0.05 μg/mL.

### 2.4. Sample Preparations

300 μL of IS working solution was added into a 96-well protein precipitation plate, followed by adding 50 μL of BALF samples, calibration standards, or QC samples. The samples were mixed on a 96-well vortex mixer (SCI-M, Scilogex, Rocky Hill, CT, USA) at 600–800 rpm for 5 min. After that, the plate was processed under a constant positive pressure of 0.04 MPa for 3 min to obtain the filtrate. Subsequently, 100 μL of the filtrate was transferred and diluted with 400 μL of 0.3% FA in water. The final solutions were analyzed by LC-MS/MS.

### 2.5. Method Validation

The LC-MS/MS assay for eravacycline quantification in BALF was validated in accordance with ICH M10: Bioanalytical Method Validation and Study Sample Analysis. The calibration ranges were 1 to 200 ng/mL for BALF. Precision and accuracy were evaluated at four levels—the lower limit of quantification (LLOQ), QCL, QCM, and QCH. For each concentration level, five replicates were analyzed using a weighted calibration curve, and the procedure was repeated across three separate analytical runs on two separate days.

Matrix effects and extraction recovery were evaluated at selected QC levels in accordance with regulatory guidelines. For the matrix effect assessment, QC samples at QCL and QCH concentrations were analyzed. BALF samples were obtained from six HAP/VAP patients without prior exposure to eravacycline. All blank BALF samples were collected from patients with HAP/VAP after their informed consent was obtained. The normalized matrix factor (MF) was determined by dividing the analyte-to-IS peak area ratio obtained from BALF samples by that from neat solutions. Extraction recovery was assessed at QCL, QCM, and QCH levels by comparing the peak areas of eravacycline in pre-spiked QC samples (samples spiked before extraction) with those in post-extraction blank BALF matrices that were spiked with the same analyte concentrations after protein precipitation. Six replicates were analyzed at each concentration level. For each individual matrix source/lot evaluated, the accuracy should be within ±15% of the nominal concentration, and the precision (expressed as percent coefficient of variation, CV%) should not exceed 15%. At all concentration levels, the CV% for the extraction recovery of the analyte did not exceed 15.0%.

Stability of eravacycline in BALF was assessed at QCL and QCH concentrations under various conditions, including room temperature, long-term storage at −20 °C and −70 °C, and three freeze–thaw cycles. In addition, post-preparative stability in the autosampler was examined.

### 2.6. Calculation of Eravacycline Concentration in Lung Epithelial Linings Fluid

During bronchoalveolar lavage, drug concentrations in BALF were diluted by the lavage fluid. Urea is a small polar endogenous molecule which can freely diffuse across the blood-bronchoalveolar barrier and rapidly equilibrate between plasma and ELF [19,23]. It is generally assumed that the urea concentration in plasma is equal to that in ELF, as shown in Formula (1):

(1) $${Urea}_{Blood}={Urea}_{ELF}$$

The dilution factor (DF) of the drug in BALF, which reflects the ratio of BALF volume to ELF volume, can be estimated by the ratio of urea concentrations in plasma and BALF (Formula (2)):

(2) $$\frac{V_{BALF}}{V_{ELF}}=\frac{{Urea}_{Blood}}{{Urea}_{BALF}}$$

The drug concentration in ELF can then be calculated by multiplying the measured concentration in BALF by the dilution factor, as shown in Formula (3):

(3) $$C_{ELF}=C_{BALF}\times \frac{V_{BALF}}{V_{ELF}}$$

Combining Formulas (3) and (4), the final equation used for calculating the concentration of eravacycline in ELF is:

(4) $$C_{ELF}=C_{BALF}\times \frac{{Urea}_{Blood}}{{Urea}_{BALF}}$$

Urea concentrations in plasma and BALF were quantified using a validated LC-MS/MS method previously established by our research group [24]. The analysis was performed on an 4500MD mass spectrometer (AB Sciex, Framingham, MA, USA) equipped with a TurboIonSpray™ source, operating in positive electrospray ionization (ESI) mode. Chromatographic separation was carried out on an Atlantis^®^ HILIC Silica column (3 μm, 2.1 × 50 mm; Waters, Ireland).

### 2.7. Clinical Applications

The method was applied in a prospective randomized clinical study to quantify eravacycline concentrations in ELF, thereby evaluating its pulmonary penetration in HAP and VAP patients. The study is registered at chictr.org.cn (ChiCTR2400087137) on 22 July 2024, and the protocol was reviewed by the Ethics Committee of Huashan Hospital, Fudan University (Approval No. 2024M-011) on 12 July 2024. Written informed consent was obtained from all participants prior to their enrollment in the study.

Eligible patients with HAP or VAP were randomized into four groups. All participants received intravenous XERAVA^TM^ (eravacycline for injection, provided by Everest Medicines Co., Shanghai, China) at a dose of 1 mg/kg every 12 h, with each infusion lasting approximately 60 min. Prior to treatment initiation, sputum specimens were collected for bacterial culture and identification, and the minimum inhibitory concentrations (MICs) of the isolated pathogens were determined using Etest (Liofilchem, Italy) on Mueller–Hinton agar, to confirm the susceptibility of the pathogens to eravacycline. Sampling was conducted following the seventh dose, as steady-state pharmacokinetics of eravacycline are anticipated to be attained based on its established PK profile [25]. Each participant underwent a single bronchoscopy with bronchoalveolar lavage (BAL) at a specific time point (2, 4, 7, or 13 h after the fifth dose infusion) assigned according to the randomization group. BAL sampling time points were selected based on the three-compartment PK characteristics of eravacycline [26]. Concurrently with BALF collection, plasma samples were collected at the same time as BALF collection to determine drug concentrations for the subsequent calculation of the ELF/plasma drug penetration ratio. The plasma and BALF samples were centrifuged and subsequently stored at −70 °C before analysis. The LC-MS/MS method developed in this study was subsequently applied for the quantification of eravacycline in BALF samples, and was also suitable for plasma sample analysis.

## 3. Result

### 3.1. Result of Method Validation

#### 3.1.1. Assay Specificity and Linearity

**Figure 1:**
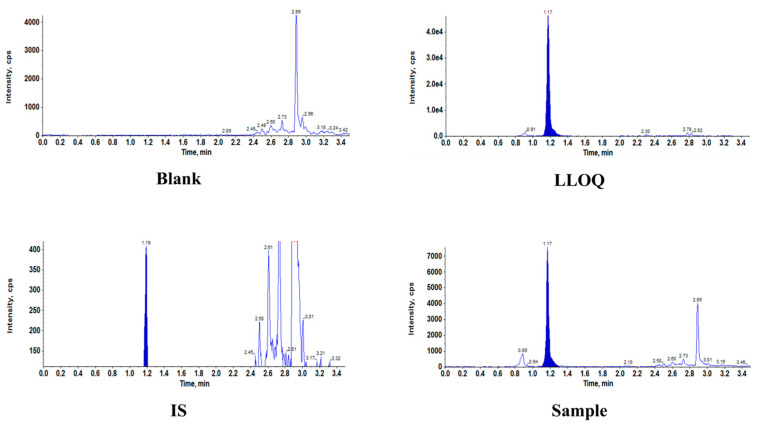
Representative chromatograms of eravacycline (ERA) and internal standards ([^2^H_8_]-ERA) in BALF. BALF, bronchoalveolar lavage fluid; IS, internal standard; LLOQ, lower limit of quantitation.

Representative chromatograms of BALF samples (with or without internal standard), LLOQ samples, and clinical samples are shown in Figure 1. The chromatograms show well-resolved peaks for eravacycline under the described chromatographic conditions, with no observable interference from endogenous matrix components on either the analyte or the internal standard channels. To assess potential interference from endogenous components, six blank BALF samples from HAP/VAP patients were analyzed. The method demonstrated high specificity, as indicated by the low background noise in blank samples from various sources and there were no interferences for the analytes and the IS either. The retention time of eravacycline and the IS eluted at approximately 1.17 min.

The typical equations of the calibration curves for eravacycline are shown in Table 1. The calibration curves exhibited good linearity over the concentration ranges of 1–200 ng/mL, with correlation coefficients (R^2^) exceeding 0.99.

#### 3.1.2. Accuracy and Precision

The accuracy and precision results for eravacycline in BALF samples are summarized in Table 2. For BALF samples, normal saline (NS) was used as a surrogate matrix for the preparation of calibration standards and QC samples for the validation of accuracy and precision. The intra-day and inter-day precision values ranged from 1.4% to 6.0% and from 1.6% to 9.9%, respectively. The intra-day and inter-day accuracy values ranged from 98.0% to 101.5% and from 99.5% to 102.4%, respectively. All values met the generally accepted criteria, with precision ≤12.2% and accuracy within ±7.5% of the nominal concentration.

#### 3.1.3. Matrix Effect and Extraction Recovery

The results of matrix effect and extraction recovery are summarized in Table 3. The MFs of eravacycline in BALF ranged from 97.4% to 107.6%. The matrix effect was assessed using six individual BALF lots, and the accuracy and precision (CV%) at each QC concentration level were within ±6.8% of the nominal values, in accordance with current regulatory guidelines. The average extraction recovery rate of eravacycline in BALF ranged from 103.5% to 107.2%, respectively. The CV% for the extraction recovery of the analyte did not exceed 13.0%.

#### 3.1.4. Stability

The stability of stock solutions was evaluated after being stored at −70 °C and room temperature. The peak areas were compared with those in fresh prepared stock solutions. The results showed that the stock solution of eravacycline was stable for 130 days at −70 °C, and for 4 h at room temperature, respectively. The stability results of spiked BALF were demonstrated in Table 4. Eravacycline in BALF showed stability for 6 h at room temperature (25 ± 2 °C) and maintained stability through three complete freeze–thaw cycles (−70 ± 10 °C to room temperature). Long-term storage stability of eravacycline in BALF was demonstrated for 44 days at both −70 ± 10 °C and −20 ± 10 °C. Additionally processed samples remained stable in the LC-MS/MS autosampler at 4 °C for up to 4 h.

### 3.2. Clinical Application

The method was employed to evaluate eravacycline concentrations in ELF in four patients with HAP or VAP following intravenous administration during a clinical study.

**Figure 2:**
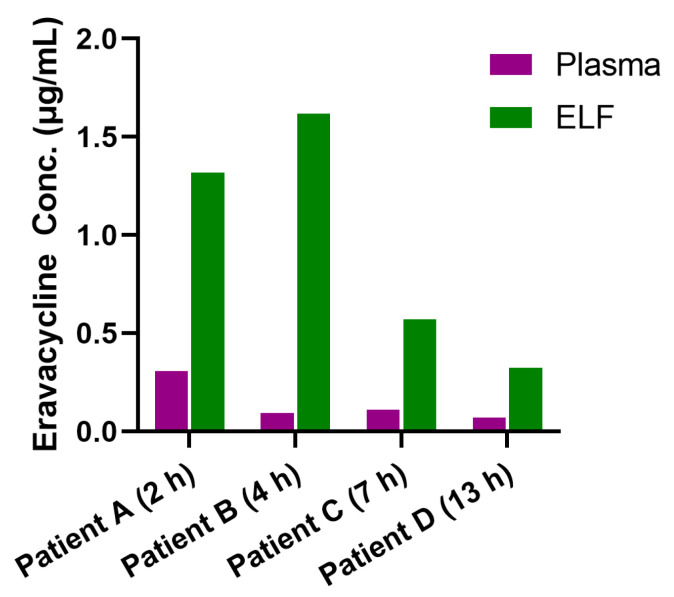
Eravacycline Concentrations in Plasma and ELF of HAP or VAP Patients After Intravenous Administration. Eravacycline concentrations in plasma and ELF, which were collected at specific time points after drug administration (2 h for Patient A, 4 h for Patient B, 7 h for Patient C and 13 h for patient D), following at least five intravenous doses of eravacycline (1 mg/kg every 12 h), each administered approximately 60 min. ELF concentrations were normalized using urea to reflect actual drug levels in pulmonary epithelial lining fluid. ELF, pulmonary epithelial lining fluid; Conc.†, concentration.

The study enrolled ICU patients with multidrug-resistant Gram-negative bacterial infections, primarily caused by CRAB or Carbapenem-resistant *Klebsiella pneumoniae* (CRKP). The demographic and clinical baseline characteristics of the study population are shown in Table S1. The MIC values of the pathogens isolated from our patients, which are shown in Table S2 (0.064–0.38 μg/mL), fall within the ranges reported in previous surveillance studies and reviews. [10,27,28] The ELF concentrations and pulmonary penetration ratios for the four patients are shown in Figure 2 and Table 5. Bronchoscopy with BAL was performed in these patients at 2 h, 4 h, 7 h and 13 h after administration of at least five doses of eravacycline, with urea-normalized ELF concentrations of 1.318, 1.617, 0.570 and 0.323 μg/mL, respectively. The pulmonary penetration ratios of eravacycline in the four patients were 4.29 (1.318 μg/mL vs. 0.307 μg/mL), 17.40 (1.617 μg/mL vs. 0.0929 μg/mL), 5.22 (0.570 μg/mL vs. 0.109 μg/mL), and 4.70 (0.323 μg/mL vs. 0.0687 μg/mL), calculated as the ratio of urea-normalized ELF concentration to plasma concentration at the corresponding time points.

## 4. Discussion

Given the physicochemical properties of eravacycline and the complexity of BALF matrices, this study therefore adopted a series of strategies to effectively establish a robust LC-MS/MS method. Firstly, protein precipitation was selected as the sample preparation strategy for eravacycline quantification, offering greater simplicity, shorter processing time, and higher analyte recovery compared to the solid-phase extraction methods as used in a previous study [16]. To further streamline sample preparation, a 96-well protein precipitation plate with vacuum-assisted filtration was employed in place of conventional centrifugation. This approach not only retains the inherent benefits of protein precipitation but also significantly enhances operational efficiency by reducing batch processing time through parallelized vacuum filtration, minimizing manual handling variability via standardized plate-based workflow, and ensuring inter-batch consistency through uniform filtration pressure control [29]. The use of a filtration-based protein precipitation plate further improves removal of precipitated proteins and particulates, enhancing sample cleanliness and consistency. This reduces ion suppression and supports reliable LC-MS/MS analysis in BALF samples [30]. During the optimization of the precipitation process, methanol was identified as the optimal precipitating solvent compared to acetonitrile, providing efficient protein removal while better preserving the chemical stability of eravacycline by avoiding rapid precipitation-induced pH shifts that can occur with acetonitrile. Secondly, TFA in mobile phase may cause ion suppression in positive-ion mode and may completely inhibit ionization in negative-ion mode due to its strong ion-pairing and surface activity effects [31]. Therefore, 0.3% FA in water was adopted as the aqueous phase modifier, which improved MS signal reproducibility. Compared to the previously reported method [16], our approach achieves improved sensitivity and a reduced run time. Collectively, these strategies enabled the development of a robust and practical method, laying a solid foundation for subsequent method validation and clinical application.

Quantifying antibiotic concentrations in the ELF, the site most representative of drug exposure at the pulmonary infection interface, is essential for evaluating therapeutic efficacy in pneumonia and for calculating pulmonary penetration ratios [18]. In this study, we describe a validated method for quantifying eravacycline concentrations in ELF. During method development for BALF samples, due to the limited availability of blank BALF from patients, NS was used as a surrogate matrix. The absence of significant interference from endogenous substances in the quantification of eravacycline and its internal standard confirmed the suitability of NS as a feasible alternative. Moreover, the use of a surrogate matrix for calibration and QC sample preparation not only simplified the analytical process for BALF samples but also enhanced the method’s practicality and efficiency. To accurately estimate ELF drug concentrations, urea is commonly used as an internal calibration factor to adjust for the dilution effect of BALF samples, thereby correcting for sample dilution during BAL [23,24]. This correction is vital for determining true target site exposure. The use of our previously developed LC-MS/MS method for urea quantification ensured accurate and precise measurement of this critical correction factor [24]. This combined approach enabled the evaluation of pulmonary penetration of eravacycline and provided valuable PK data in the infection site, which is particularly relevant for treating pneumonia such as HAP and VAP.

However, while urea remains the most commonly used marker, its validity may be affected in patients with renal dysfunction or systemic inflammation due to alterations in systemic and alveolar urea dynamics. [32,33]. For example, Yu et al. reviewed ICU-admitted patients undergoing BAL and reported a median plasma-to-BALF urea ratio of 4.2 (IQR 3.2–8.6), illustrating variability in BALF dilution among critically ill populations, which may be influenced by inflammation, oxygenation, and pulmonary mechanics [34]. In addition, Rennard et al. reported that urea concentrations may increase during the course of BAL, potentially leading to biased estimates of ELF dilution [35]. Nonetheless, in the absence of a validated alternative, urea-based correction remains the most practical and widely accepted approach for estimating ELF dilution during BAL, with procedural measures such as minimizing dwell time helping to mitigate potential concerns [36].

Very limited PK data are available from critically ill patients, who are both most affected by MDR microorganisms and most likely to benefit from novel antibacterial agents [37]. The necessity of conducting PK studies in this population is underscored by the profound PK alterations that frequently occur in the context of multi-organ dysfunction [17]. Such changes can lead to subtherapeutic antimicrobial exposure, reduced efficacy, and an increased risk of toxicity. Within this setting, the development of a robust and sensitive LC-MS/MS method becomes especially critical. This analytical platform not only enables precise quantification of drug concentrations in plasma and ELF but also establishes a methodological basis for therapeutic drug monitoring (TDM) in real-world ICU environments. By providing accurate measurement of eravacycline concentrations, LC-MS/MS supports the assessment of target attainment across a range of pathogen MICs, aids in dose optimization for patients with altered PK, and helps elucidate exposure–response relationships. Ultimately, integrating validated LC-MS/MS assays into clinical PK/PD studies may promote more individualized and evidence-based antimicrobial therapy in critically ill patients.

Furthermore, the validated LC-MS/MS method was successfully applied to quantify eravacycline concentrations in ELF from four patients with HAP or VAP in a prospective study. Despite the small sample size, this clinical application demonstrated the method’s practicality and robustness in a real-world setting. The pulmonary penetration ratio was calculated as the ratio of the urea-corrected ELF concentration to the simultaneously measured plasma concentration in this study, serving as a surrogate indicator for drug distribution in lung tissues. Considerable inter-individual variability was observed, which may be related to differences in pulmonary permeability, local inflammation, and protein binding in these critically ill patients [38]. In addition, the PK/PD index most relevant to eravacycline efficacy is the ratio of the free area under the concentration–time curve to the minimum inhibitory concentration (*f*AUC/MIC). However, due to ethical restrictions and concerns regarding patient tolerance, each participant in our study underwent only one BAL. As a result, the dynamic changes in ELF concentrations over time and the calculation of individual AUC values and detailed probability of target attainment (PTA) simulations could not be assessed. Although current guidelines do not recommend a loading dose for eravacycline, it remains uncertain whether such a strategy could enhance early antibiotic efficacy—as is the case with tigecycline or β-lactams in the context of severe septic shock or severely immunocompromised patients [39,40]. Our investigation was specifically designed to assess pulmonary penetration under the currently approved dosing scheme in China and thus did not evaluate the potential utility of a loading dose. Further PK and clinical studies are needed to determine whether an optimized dosing strategy, potentially incorporating a loading dose, could improve eravacycline’s therapeutic performance in this vulnerable patient population. To ensure effective management of pulmonary infections, combination therapy, including polymyxin B, ceftazidime-avibactam, and other antibiotics, was administered in certain cases, building on in vitro confirmation of eravacycline susceptibility. The impact of such combination regimens on pharmacokinetics, safety, and efficacy will be further evaluated in future studies using population pharmacokinetic (PPK) modeling. Despite this limitation, this study provides preliminary data on the intrapulmonary distribution of eravacycline in patients with pneumonia, supporting its potential utility in the treatment of HAP and VAP. Future studies with larger cohorts and expanded sampling time points are needed to better characterize the pulmonary pharmacokinetics of eravacycline and to clarify its correlation with clinical outcomes. Nevertheless, this LC-MS/MS method may serve as a practical tool for future therapeutic drug monitoring and PK/PD study of eravacycline in critically ill patients.

## 5. Conclusions

In this study, a simple, rapid, and sensitive LC-MS/MS method was developed for quantifying eravacycline in BALF. With a 3.5 min run time per injection and minimal sample preparation, the method is well-suited for high-throughput clinical analysis. Notably, this is the first study to report the pulmonary penetration of eravacycline in patients with HAP or VAP. The validated method was successfully applied to clinical BALF samples, supporting its utility in PK and pulmonary distribution studies.

## Figures and Tables

**Table 1:** Calibration curve parameters for eravacycline from four different batches in BALF.

Analyte	Batch	Slope	Intercept	R^2^	Fitted Equation
Eravacycline in BALF	1	0.00421	0.00156	0.99870	y = 0.00421x + 0.00156
	2	0.00550	0.00172	0.99900	y = 0.0055x + 0.00172
	3	0.00353	0.00106	0.99850	y = 0.00353x + 0.00106
	Average	0.0044	0.0014	0.9987	

BALF, bronchoalveolar lavage fluid.

**Table 2:** Intra- and inter-day precision and accuracy values of eravacycline in BALF.

Samples	Nominal Conc.† (ng/mL)	Average Measured Conc.† (ng/mL)	Precision (%)	Accuracy (%)
Intra-batch (*n* = 5)				
LLOQ	1	1.015	1.6	101.5
QCL	3	2.970	6.0	99.0
QCM	60	59.460	2.4	99.1
QCH	150	147.000	1.4	98.0
Inter-batch (*n* = 15)				
LLOQ	1	1.024	9.9	102.4
QCL	3	3.018	4.4	100.6
QCM	60	61.380	4.0	102.3
QCH	150	149.200	1.6	99.5

Conc.†: Concentration; *n*: Replicate; LLOQ, lower limit of quantitation; QCL, low quality control; QCM, medium quality control; QCH, high quality control; BALF, bronchoalveolar lavage fluid. Precision (%) = (standard deviation/mean measured concentration) × 100%; Accuracy (%) = (mean measured concentration/nominal concentration) × 100%.

**Table 3:** Matrix effect and extraction recovery of eravacycline in human BALF samples.

QC Samples	BALF (*n* = 6)		
	Average (%)	SD	CV (%)
Matrix effect (%)			
QCL	97.4	6.6	6.8
QCH	107.6	2.7	2.5
Extraction recovery (%)			
QCL	103.5	/	6.1
QCM	103.6	/	13.0
QCH	107.2	/	1.4

BALF, bronchoalveolar lavage fluid; QCL, low quality control; QCM, medium quality control; QCH, high quality control; SD, standard deviation; CV, coefficient of variation.

**Table 4:** The stability of eravacycline in BALF.

Condition	QCL		QCH	
	Recovery (%)	CV (%)	Recovery (%)	CV (%)
Room temperature 6 h	112.1	4.8	107.6	0.9
Post-preparation (autosampler, 4 °C) 4 h	91.8	1.8	96.3	4.4
Three freeze–thaw cycles (−70 °C)	107.8	7.8	98.4	2.1
Long-term 44 days (−20 °C)	107.1	3.2	105.3	3.0
Long-term 44 days (−70 °C)	110.4	3.3	107.5	2.1

BALF, bronchoalveolar lavage fluid; QCL, low quality control; QCH, high quality control; CV, coefficient of variation.

**Table 5:** ELF concentration and pulmonary penetration ratios of eravacycline after multiple intravenous doses at scheduled time points.

Patient ID	Time (h)	Urea_Blood_/Urea_BALF_	Eravacycline Conc.† (μg/mL)			Pulmonary Penetration Ratios
			Plasma	BALF	ELF	
A	2 h	30.27	0.307	0.0436	1.318	4.29
B	4 h	69.10	0.0929	0.0234	1.617	17.40
C	7 h	16.98	0.109	0.0336	0.570	5.22
D	13 h	15.23	0.0687	0.0212	0.323	4.70

BALF, bronchoalveolar lavage fluid; ELF, pulmonary epithelial lining fluid; Conc.†, concentration. Pulmonary penetration ratio (%) = ELF concentration/plasma concentration × 100%.

## Data Availability

The data supporting the findings of this study are available from the corresponding author, Professor Jing Zhang, upon reasonable request.

## Supplementary Materials

The following supporting information can be downloaded at: https://www.mdpi.com/article/10.3390/antibiotics14090957/s1, Table S1: Demographic and clinical baseline characteristics of the study population; Table S2: Primary disease and pathogenic information of the study population.

## Abbreviations

The following abbreviations are used in this manuscript:

HAP	Hospital-acquired pneumonia
VAP	Ventilator-associated pneumonia
ELF	Pulmonary Epithelial lining fluid
PK	Pharmacokinetic
PK/PD	pharmacokinetic/pharmacodynamic
BALF	Bronchoalveolar lavage fluid
ICU	Intensive care units
MDR	Multidrug-resistant
CRAB	Carbapenem-resistant *Acinetobacter baumannii*
CRKP	Carbapenem-resistant *Klebsiella pneumoniae*
XDR	Extensively drug-resistant
CRE	Carbapenem-resistant *Enterobacteriaceae*
cIAI	Complicated intra-abdominal infections
TFA	Trifluoroacetic acid
MRM	Multiple reaction monitoring
NS	Normal saline
LLOQ	Lower limit of quantification
MF	Matrix factor
DF	Dilution factor
ESI	Electrospray ionization
BAL	Bronchoalveolar lavage
TDM	therapeutic drug monitoring
fAUC/MIC	free area under the concentration–time curve to the minimum inhibitory concentration
PTA	probability of target attainment
PPK	population pharmacokinetic
