# Supplementary material for: Development and Validation of a Rapid LC-MS/MS Method for Quantifying Eravacycline in Epithelial Lining Fluid: Application to a Prospective Pulmonary Distribution Study in HAP/VAP Patients
Source: Antibiotics (Basel). 2025 Sep 22;14(9):957. doi: 10.3390/antibiotics14090957 (PMC12466610; doi:10.3390/antibiotics14090957)
Supplement: Supplementary file 1 [file antibiotics-14-00957-s001.zip › antibiotics-3833175-supplementary.pdf]

Supplemental Appendix

Table S1. Demographic and clinical baseline characteristics of the study population.

	**ERV (n=4)**
**Sex—no. (%)**	Male (100%)
**Age—yr**	56 (19)
**BMI (kg/m^2)**	24.5 (4.7)
**CPIS**	7.3 (0.9)
**APAECH II score**	18.5 (5.1)
**Body temperature**	37.6 (0.5)
**WBC（*10^9/L）**	11.25 (3.40)
**CRP（mg/L）**	61.5 (66.5)
**Scr (μmol/L)**	76 (41.5)
**CrCl (mL/min)**	99.6 (34.6)

No., number; ERV, eravacycline; BMI, Body Mass Index; CPIS: clinical pulmonary infection score; APAECH II, acute physiology and chronic health evaluation II; WBC, white blood cell; CRP, C-reactive protein; Scr, serum creatinine; CrCl, creatinine clearance rate.

Table S2. Primary disease and pathogenic information of the study population.

	**Patient A**	**Patient B**	**Patient C**	**Patient D**
**Primary Diseases**	Diabetes Mellitus, Respiratory Failure Type 1	Traumatic Intracranial Hemorrhage, Traumatic Cerebral Infarction	Malignant Neoplasm of Brain Stem, Hydrocephalus	Mild Closed Craniocerebral Injury, Hydrocephalus
**Pathogen （MIC μg/ml）**	CRAB (0.38); CRKP (0.25)	CRAB (0.38)	CRAB (0.064)	CRAB (0.25)

MIC, Minimum Inhibitory Concentration, MIC values were determined using the Etest; CRAB, Carbapenem-resistant *Acinetobacter baumannii*; CRKP, Carbapenem-resistant *Klebsiella pneumoniae*.
